# Can acupuncture improve sleep quality and anxiety among women during perimenopause? A protocol for systematic review and meta-analysis

**DOI:** 10.1097/MD.0000000000028449

**Published:** 2022-01-14

**Authors:** Yanpei Ping, Chao Liang, Xixi Fan, Lili Zhang, Dashi Ying, Zhongnan Wang

**Affiliations:** a Changchun University of Chinese Medicine, Changchun, China.; b The Affiliated Hospital of Changchun University of Chinese Medicine, Changchun, China.; c Jilin Agricultural Science and Technology University, Jilin, China.

**Keywords:** acupuncture, anxiety, perimenopause, sleep, systematic review

## Abstract

**Background:**

The decrease in estrogen levels during the perimenopausal period can cause women to have various symptoms such as insomnia, emotional anxiety, and even depression. Therefore, whether the green therapy of acupuncture can improve the sleep quality and anxiety of perimenopausal women has attracted more and more attention. The purpose of this systematic evaluation was to assess the efficacy of acupuncture on insomnia and anxiety in perimenopausal women.

**Methods:**

We will search for clinical observational pilot studies or cohort studies of acupuncture for insomnia, anxiety, or depression included in PubMed, Cochrane Library, Embase, Web of science, China Knowledge Network (CNKI), Wanfang, VIP and China Biomedical Database (CBM), etc. The search period will be from the establishment of the database until November 2021. Two researchers will independently perform literature screening, data extraction, and quality assessment. Finally, data analysis will be performed using Revman and Stata software.

**Results:**

The purpose of this study was to evaluate the effectiveness and safety of acupuncture therapy for the treatment of insomnia, anxiety, and depression in perimenopausal women.

**Conclusion:**

This study will provide new evidence on the effectiveness and safety of acupuncture for the treatment of insomnia, anxiety, and depression in perimenopausal women, and provide additional options for clinicians and patients to improve insomnia and anxiety.

**Registration Number:**

INPLASY2021120012

## 1 Introduction

Insomnia remains one of the most common sleep disorders among perimenopausal women and is usually characterized by subjective complaints of difficulty falling asleep or maintaining sleep, or interrupted sleep, or non-recovery of sleep, producing significant daytime symptoms, including poor concentration and mood disturbances.^[1]^ Insomnia is a common sleep disorder that is associated with a range of adverse outcomes.^[2]^ Insomnia is common in perimenopausal women, increasing the risk of depression in these already vulnerable people. Anxiety symptoms increase during the perimenopausal period, and subclinical depressive symptoms can also increase the risk of severe depression. The risk of depressive episodes increases during the perimenopausal period, and women experiencing menopausal symptoms report elevated levels of depressive symptoms. Insomnia symptoms are one of the most common complaints of perimenopausal women. Nearly half of women (43–48%) have insomnia symptoms.^[3]^ However, given the high comorbidity of insomnia and depression,^[4,5]^ even with a common etiology,^[6,7]^ the gold standard therapy for the treatment of perimenopause-related sleep disorders should also ideally alleviate co-occurring depressive symptoms and depressive behaviors in perimenopausal women. Chronic insomnia, lack of adequate sleep, and anxiety are also considered to be risk factors that contribute to medical problems such as cardiovascular disease, diabetes, obesity, and asthma, which emphasizes the necessity of identifying and treating insomnia.^[8,9]^ Insomnia is often comorbid with depression, and there is a bidirectional relationship between these disorders. It is not surprising that there is universal interest in finding effective complementary and alternative therapies to treat insomnia and anxiety with a lower risk of adverse effects or withdrawal.^[10]^ There is evidence^[11–18]^ that interventions targeting insomnia, such as acupuncture therapy, may lead to improvements in insomnia and depression. The purpose of this systematic evaluation and meta-analysis was to determine the efficacy of acupuncture in treating insomnia and anxiety and depression.

## 2 Why it is significant to carry out this review

Sleep deprivation as well as emotional anxiety are major sources of harmful diseases related to heart, brain, psychological changes, hypertension, diabetes, weight gain, etc. 40% to 50% of the world's population suffers from sleep deprivation. Considering the interaction between sleep disorders and anxiety and depression, as well as the evolution of disease and the increased risk of insomnia-related comorbidities, it is important to diagnose and treat insomnia and its comorbidities. Acupuncture, as a green therapy in addition to medication, is a new expectation for our treatment of insomnia, and there is no systematic review to evaluate the efficacy of acupuncture in improving sleep quality and relieving anxiety. Therefore, a comprehensive review of perimenopausal female patients would provide patients and clinicians with an analysis of the overall effectiveness.^[8]^

## 3 Objectives

The decrease in estrogen levels during the perimenopausal period can cause women to have various symptoms such as insomnia, emotional anxiety, and even depression. Therefore, whether the green therapy of acupuncture can improve the sleep quality and anxiety of perimenopausal women has attracted more and more attention. The purpose of this systematic review is to evaluate the efficacy of acupuncture.

## 4 Methods

The protocol has been registered in INPLASY (INPLASY2021120012), and completed according to the preferred reporting items for systematic reviews and meta-analysis protocol (PRISMA-P).^[19]^ Should any amendments to this protocol be necessary, they will be documented on the INPLASY platform.

### 4.1 Criteria for including studies in this review

#### 4.1.1 Types of studies

This study will include randomized controlled trials of acupuncture for insomnia, anxiety, and depression, either by blinded or allocation concealment methods. The language of this study's included literature is limited to English or Chinese.

#### 4.1.2 Types of participants

Perimenopausal women are clearly identified by clinicians as chronic sleeplessness accompanied by emotional anxiety. The diagnostic criteria for insomnia, anxiety, and depression are not subject to any restrictions. There are no restrictions on gender, race, or source of cases.

#### 4.1.3 Types of interventions

The intervention treatment group included traditional acupuncture, warm acupuncture, and electric acupuncture. The control group included Drug therapy, Chinese herbal medicine, music therapy, cognitive–behavioral therapy (CBI), and placebo.

#### 4.1.4 Comparator(s)/control

The intervention treatment group included traditional acupuncture, warm acupuncture, and electric acupuncture. The control group included Drug therapy, Chinese herbal medicine, music therapy, cognitive–behavioral therapy (CBI), and placebo.

### 4.2 Outcomes

#### 4.2.1 Primary outcomes

The total clinical effectiveness rate was used to observe the efficacy of acupuncture in treating insomnia and depression, and the total clinical effectiveness rate was used as the primary outcome index.

#### 4.2.2 Secondary outcomes

The Pittsburgh Sleep Quality Index was used to assess the sleep quality of perimenopausal women. Hamilton Anxiety Inventory and Hamilton Depression Inventory were used to assess patients’ anxiety and depression indices.

### 4.3 Search methods for identification of studies

#### 4.3.1 Searching from following databases

We will search for clinical observational pilot studies or cohort studies of acupuncture for insomnia, anxiety, or depression included in PubMed, Cochrane Library, Embase, Web of science, China Knowledge Network (CNKI), Wanfang, VIP, and China Biomedical Database (CBM), etc. The search period will be from the establishment of the database until November 2021. In this study, a combination of MESH subject terms and free words was used for literature search, and the search languages included English and Chinese. The search strategy for PubMed is shown in Table 1. The search strategies for other databases were approximately the same.

**Table 1:** The search strategy for PubMed database.

No.	Search items
#1	“Sleep Initiation and Maintenance Disorders”[MeSH Terms]
#2	“insomnia”[Title/Abstract]OR“sleeplessness”[Title/Abstract]OR“sleep disorders”[Title/Abstract] OR “dyssomnia”[Title/Abstract]
#3	#1 OR #2
#4	“anxiety”[MeSH Terms]
#5	“Angst”[Title/Abstract]OR“Nervousness”[Title/Abstract]OR“Hypervigilance”[Title/Abstract] OR “Anxiousness”[Title/Abstract]
#6	#4 OR #5
#7	“depressive disorder”[MeSH Terms] OR “depression”[MeSH Terms]
#8	“Depressions”[Title/Abstract] OR “depressive symptoms”[Title/Abstract] OR “depressive symptom”[Title/Abstract]
#9	#7 OR #8
#10	#3 AND #6 OR #9
#11	“acupuncture”[MeSH Terms] OR “acupuncture therapy”[MeSH Terms]
#12	“acupuncture therapy”[Title/Abstract] OR “Electroacupuncture”[Title/Abstract] OR “warm acupuncture”[Title/Abstract]
#13	#11 OR #12
#14	Randomized controlled trail[MeSH Terms]
#15	(((RCT[Title/Abstract]) OR (Clinical trail[Title/Abstract])) OR (Randomized clinical trail[Title/Abstract])) OR (cohort study[Title/Abstract])
#16	#14 OR #15
#17	#10 AND #13 AND #16

RCTs = randomized clinical trials.

#### 4.3.2 Searching by other approaches

To obtain more comprehensive search results, we will also search the US Clinical Trials Registry and the Chinese Clinical Trials Registry for ongoing or incomplete clinical trials.

### 4.4 Data collection and analysis

#### 4.4.1 Selection of studies

**Figure 1:**
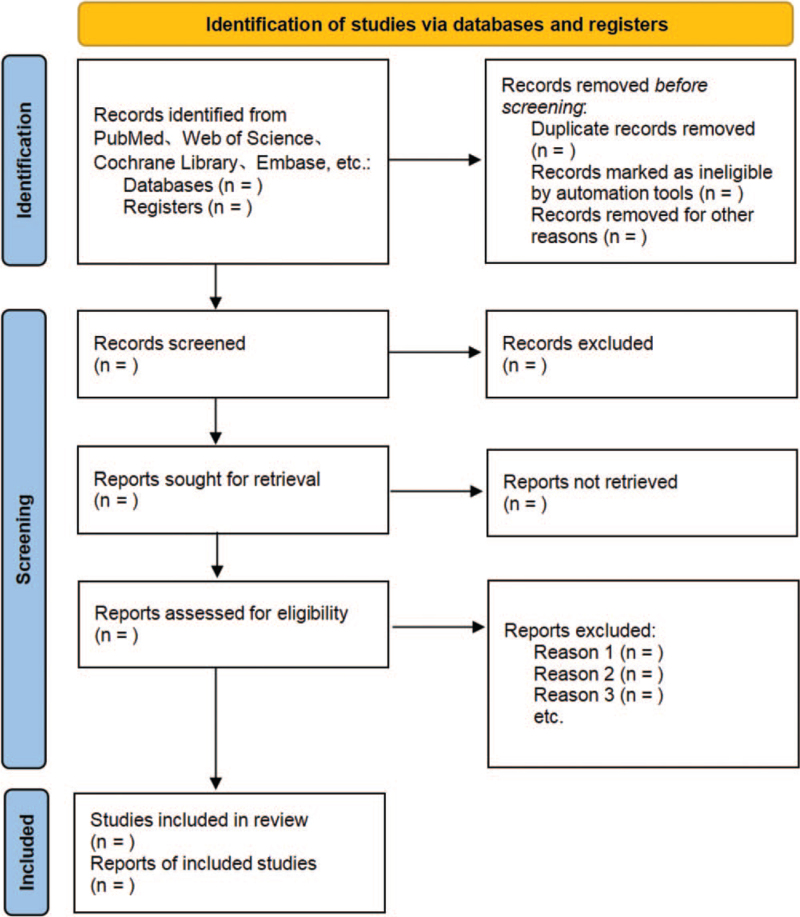
The PRISMA flow chart. Flow diagram of study selection process. PRISMA = preferred reporting items for systematic reviews and meta-analysis.

All titles and abstracts for this study search will be independently screened by 2 researchers based on inclusion criteria. The full text of eligible articles will be reviewed when necessary. The procedure of studies selection is shown in a Preferred Reporting Items for Systematic Reviews and Meta-Analyses diagram Fig. 1.

#### 4.4.2 Extraction and management of date

Two researchers were selected to independently search Chinese and English databases to obtain literature that met the requirements, and then imported into EndNote X9 software for literature screening, first reading the titles and abstracts of the literature, excluding literature that did not meet the inclusion criteria, reading the full text of the literature that was initially determined to meet the inclusion criteria, eliminating duplicate literature, incomplete data and literature with unreasonable experimental design, and extracting data including Author's name, publication time, trial method, sample size, patient gender, age, disease duration, interventions, and outcome indicators. Two researchers independently screened and extracted the above data, and then cross-checked the data. In case of disagreement, the third researcher led the discussion and joint discussion, and finally determined the literature to be included in the Meta-analysis.

#### 4.4.3 Evaluating risk of bias

We will use RevMan software to evaluate the quality of the literature by the Cochrane risk bias assessment tool for 6 aspects of the included literature, including random assignment method, allocation scheme concealment, blinding, completeness of outcome data, selective reporting of study results, and other sources of bias, and the evaluation levels are divided into 3 levels: low risk bias, high risk bias, and unclear.

#### 4.4.4 Assessment of heterogeneity

First, the included literature was tested for heterogeneity, and when *P* ≥ .1 and *I*^2^ < 50% suggested no significant statistical heterogeneity, a fixed-effects model was used; when *P* < .1 and *I*^2^ ≥ 50% suggested the existence of statistical heterogeneity, if the degree of heterogeneity was within an acceptable range, a random-effects model was selected for the combined analysis, while subgroup analysis, sensitivity analysis, and Meta-regression should be performed according to the situation to explain the source of heterogeneity.

#### 4.4.5 Data synthesis and analysis

The dichotomous variables were analyzed statistically using relative risk (RR); the continuous variables were analyzed using mean standard deviation (mean difference [MD]) or weighted (SMD) as the combined effect measure, and their 95% confidence intervals were calculated.

#### 4.4.6 Assessment of publication biases

To observing the biases of potential reporting, funnel plots will be emerged when >10 studies are included.^[20]^

#### 4.4.7 Subgroup analysis and investigation of heterogeneity

In case of significant heterogeneity, we will conduct subgroup studies based on the type of insomnia, the age of the patient, and the intervention.

### 4.5 Sensitivity analysis

When the combined results of the remaining documents were not significantly different from those without deletion after the deletion of any one of them, it means that the sensitivity analysis was passed.

### 4.6 Summary of evidence

The evaluation levels are divided into 3 levels: low risk bias, high risk bias, and unclear.

### 4.7 Ethics and dissemination

The current study does not require ethical approval as all included data will be obtained from published articles and will be published in a peer-reviewed journal.

## 5 Discussion

Insomnia as a common sleep disorder with difficulty falling asleep or sleep disorder, and is accompanied by excessive dreaming and easy waking, difficulty falling asleep or seeming to fall asleep after waking, and daytime dysfunction such as dizziness, fatigue, and anxiety after waking. Insomnia is characterized by difficulty in starting sleep, maintaining sleep continuity, or poor sleep quality. Decreased estrogen levels during perimenopause can lead to a variety of symptoms in women, including insomnia, emotional anxiety, and even depression. This can lead to a range of negative emotions, such as daytime lethargy, low productivity, slower reactions, and reduced cognitive ability, while insomnia and anxiety are also considered to be risk factors contributing to medical problems such as cardiovascular disease, diabetes, obesity, and asthma, adding to the public health burden on families and society.^[21]^ Currently, most of the medications used to treat insomnia and anti-anxiety can cause some side effects to patients, such as headache, dizziness, and fatigue. So, finding effective complementary and alternative therapies for insomnia and anxiolysis and reducing the risk of adverse effects or withdrawal would be the significance of this study. Therefore, a systematic review to evaluate whether acupuncture is effective in treating insomnia and relieving anxiety and depression is highly warranted.

## Author contributions

**Conceptualization:** Yanpei Ping, Zhongnan Wang.

**Data curation:** Chao Liang, Xixi Fan.

**Formal analysis:** Chao Liang, Xixi Fan.

**Funding acquisition:** Yanpei Ping, Zhongnan Wang.

**Investigation:** Yanpei Ping, Chao Liang, Lili Zhang.

**Methodology:** Xixi Fan, Lili Zhang, Dashi Ying.

**Project administration:** Lili Zhang, Dashi Ying.

**Resources:** Xixi Fan.

**Software:** Chao Liang, Xixi Fan, Dashi Ying.

**Supervision:** Lili Zhang, Dashi Ying, Zhongnan Wang.

**Validation:** Yanpei Ping, Zhongnan Wang.

**Visualization:** Lili Zhang.

**Writing – original draft:** Yanpei Ping.

**Writing – review & editing:** Yanpei Ping, Zhongnan Wang.
